# Association Between Financial Support and Physical Health in Older People: Evidence from CHARLS Data

**DOI:** 10.3390/healthcare13101163

**Published:** 2025-05-16

**Authors:** Enkai Guo, Jing Li, Yiyuan Sun, Lan Zheng

**Affiliations:** 1 College of Physical Exercise, Hunan Normal University, Changsha 410012, China; guoenkai18@126.com; 2 Hunan Provincial Research Base for Public Service of Sports, Hunan Normal University, Changsha 410012, China; 3 Key Laboratory of Physical Fitness and Exercise Rehabilitation of Hunan Province, Hunan Normal University, Changsha 410012, China; 4 College of Physical Exercise and Sports, Beijing Normal University, Beijing 100875, China; sun14170045@163.com

**Keywords:** financial support, older people, physical health, physical exercise, social contact

## Abstract

**Background/Objectives**: Prior research has established the significant role of financial support in shaping older adults’ physical health but often overlooks the heterogeneous effects of distinct financial support types and their underlying mechanisms. This study addresses these gaps by investigating how property-based support and children’s financial support differentially influence the health of older people, aiming to inform targeted interventions for healthy aging. **Methods**: Based on 2020 microdata from the China Health and Retirement Longitudinal Study (CHARLS), the analysis was conducted using the ordered logistic regression model and the mediation effect model. **Results**: Property ownership demonstrated a significant positive association with older adults’ physical health (β = 0.21, *p* < 0.01), while children’s financial support showed an adverse effect (β = −0.14, *p* < 0.05). These relationships were mediated by two key pathways: enhanced social participation (accounting for 32%) and increased engagement in sports activities (accounting for 28%). **Conclusions**: The study underscores the need to differentiate between financial support sources when designing aging policies. Recommendations include incentivizing asset accumulation among older adults, promoting delayed retirement for capable individuals, and fostering community-based initiatives to boost social and physical activity participation. These findings advocate for integrated policy frameworks that combine financial empowerment with social engagement opportunities to address aging challenges in China.

## 1. Introduction

Population aging is a prominent trend in contemporary society, driven by enhanced quality of life and medical progress that have significantly prolonged life expectancy [1]. Conversely, economic development and pressures have impacted family fertility decisions, leading to a notable decline in birth rates. Japan, for example, has witnessed a continuous drop in its fertility rate since 1990 despite local policies aimed at boosting it. Economic considerations often prompt families to prioritize careers over child-rearing, resulting in delayed or foregone pregnancies [2]. Similarly, China is also grappling with an aging demographic, with the population aged 65 and above projected to rise from 12% in 2020 to 21% by 2035. This shift will elevate China’s old-age dependency ratio from 0.20 in 2020 to 0.39 in 2035 [3]. China’s aging index is anticipated to match Germany’s current level by 2035 and surpass the United States by 2050. The implications of population aging are profound for both the economy and society, particularly concerning national fiscal sustainability. With the growth of the aging population and the sudden decline of the young population, the older adult dependency ratio will further surge, which will pose a huge challenge to the government’s social security expenditure. In such cases, governments often resort to measures such as extending the length of employment, as in Japan, to alleviate related social and economic problems. Should financial disparities worsen, governments may either relinquish certain functions or reduce market engagement, both scenarios risking governmental and market failure alongside socioeconomic disruption.

Recently, the Chinese government released the “14th Five-Year Plan for National Economic and Social Development of the People’s Republic of China and the Outline of the 2035 Vision Goals”, which puts forward a national strategy to actively cope with population aging, aiming to improve the quality of life of the older population and promote active aging through coordinated efforts across sectors such as the economy, sports, and medical care [4]. Given the current social conditions, the economic security of older people significantly impacts their physical health [5] and comprises three main components: individual incomes (retirement wages and re-employment earnings), family economic support (financial assistance from children and relatives) and social support (social insurance and donations) [6]. In China, due to an imperfect social security system and generally low economic incomes among older people, family economic support plays an increasingly crucial role in their physical health.

This study examines the impact of financial support on the physical health of older adults in China, using micro-cross-sectional data from the China Health and Retirement Longitudinal Study (CHARLS) in 2020. The research has three main objectives: (1) to investigate the correlation between property ownership, children’s financial support, and the physical health of older individuals; (2) to determine whether these correlations are positive or negative; and (3) to explore how financial support affects physical health, considering the mediating roles of physical exercise and social participation.

## 2. Literature Review

As the population ages, addressing the challenges associated with an aging society has become a significant social issue. Amid China’s rapid urbanization and industrialization, the social economy is evolving, and the demographic structure is shifting as those who grew up during this transformative period are gradually entering old age [7]. A key objective of proactive aging policies is to improve the quality of life of older people, which is often closely linked to their economic resources, including intergenerational support, skills income, and property income. Intergenerational support plays a crucial role in the financial well-being of older people, especially in the context of traditional Chinese culture and customs, where the concept of “raising children for old age” remains prevalent, and children are seen as a vital source of support in retirement [8]. Property income, especially from house rentals, is also a significant source of income for urban older adults, often sufficient to cover their basic living expenses [6]. Under current socioeconomic conditions, with evolving life perspectives and the widespread adoption of pension insurance, some older people are able to generate income through re-employment in professions such as doctors, pharmacists, and teachers or by receiving benefits from employee retirement insurance [9].

In contrast, as a relatively stable economic source, property financial support has been found to have a significant correlation with the health of older people. Some scholars have pointed out that older people in urban areas who own fixed assets, such as real estate and shops, can obtain economic income by renting these properties, which positively impacts their health [6]. Owner-occupied housing serves not only as a residence but also as a store of wealth for older people [10]. This property income can enhance living standards, allowing older people to participate in physical exercise and afford sports consumption beyond basic needs [11], thus promoting better health outcomes. Conversely, older people without rental income or pensions and who completely rely on financial support from others may face increased medical consumption [12].

As older people age, their physical functions decline, increasing their dependence on family care and making financial support from family members particularly important for their health [6]. When children live far away from their parents or fail to provide any economic or material help, the negative impact on the health of older people is significant [13]. Unfortunately, in today’s urban settings, the high cost of living often makes it difficult for young people to allocate a substantial portion of their modest salary to support older family members, usually stepping in only during emergencies such as illness or accidents. This situation can lead to an insignificant or even negative correlation between children’s financial support and the health of older adults, particularly in rural areas [14]. The gap between urban and rural areas refers to the great difference in development between cities and rural areas due to their different geographical locations, economic bases, core industries, and human resources. With the acceleration of urbanization in China, the gap between urban and rural areas has become increasingly prominent, resulting in huge differences in the economic and health needs of older adults, which, in turn, affects the effectiveness of financial support. In addition, variations within the older population also shape different economic needs and health behaviors due to the internal age gap. For example, younger seniors aged 60–70 are often recently retired, and many have opportunities for secondary jobs or relatively abundant economic sources [15]. In contrast, older people over 70 face greater health challenges and declining opportunities to earn money, making their children’s financial support their main source of income [16]. Studies have also shown that older people receiving family financial support tend to have higher social engagement and motivation. Those with ample financial resources often have better nutrition [17], lower medical expenses, and a positive self-image, contributing to greater social confidence [18]. Encouraging older people to socialize through activities such as sports, gardening, and lectures can improve their social, physical, and mental health. To further explore how financial support affects the physical health of older people, this study introduced social participation as a mediating variable to study its action path. In addition, exercise participation, directly related to physical health, was considered another mediating variable.

Based on this, the following research hypotheses are proposed in this study:

**H1.**  *The higher the property income, the better the physical health of older people*.

**H2.**  *The financial support of children is inversely related to the physical health of older people*.

**H3a.**  *The effects of property ownership and children’s financial support on the physical health of older people vary by age group*.

**H3b.**  *The impact of property ownership and children’s financial support on the physical health of older people differs between urban and rural areas*.

## 3. Materials and Methods

### 3.1. Data Sources

This study utilizes data from the China Health and Retirement Longitudinal Study (CHARLS) database (available at https://charls.pku.edu.cn/, accessed on 19 June 2023). CHARLS aims to collect high-quality micro-data representing households and individuals aged 45 and above in China to analyze the problem of population aging in China and promote related interdisciplinary research on aging. Its baseline survey in China started in 2011, covering 150 county-level units and 450 village-level units, with a sample size of about 17,000 people in 10,000 households. The sample is tracked every 2–3 years. First, the most recent data available in the CHARLS database are from 2020. Second, despite the impact of COVID-19 in this era, the spread of the epidemic in China in 2020 has been basically effectively controlled based on China’s response speed and emergency management mechanism, and the immunization capacity of older adults has been enhanced through vaccination and other means. Finally, under the influence of the epidemic, the rise of new exercise methods such as home fitness, live fitness, and online fitness courses in China has provided a guarantee for improving the health level of older adults. Therefore, the study used StataMP 16 software to analyze a sample of 9417 older adults in 2020. In order to verify that the sample data of Chinese older people in 2020 are still representative, this study also used the data sample of 2018 in the robustness test part.

### 3.2. Variable Selection

#### 3.2.1. Dependent Variable

In this study, self-rated health status was chosen to measure the physical health of older people [19], serving as the dependent variable (Health). Participants were asked, “How do you feel about your health?”, with responses ranging from “very good” to “very poor” on a 1–5 scale, as shown in Table S1. According to the descriptive statistical results of the categorical variables in Table 1, it can be seen that nearly half of the respondents rate their physical health as average, while approximately 29% consider their physical health poor or very poor.

#### 3.2.2. Independent Variable

There are two independent variables in this study: property ownership (Property) and children’s financial support (CFS). In the subsequent robustness test, these variables were transformed to calculate whether individuals received property ownership (Property_s) or children’s financial support (CFS_s). Property ownership mainly refers to income obtained from renting fixed assets, such as real estate. As can be seen from Table 1, only 4.11% of older people in the sample have property ownership. On the contrary, around two-thirds received their children’s financial support, indicating that children’s financial support remains the primary economic source of older people in China, supplemented by real estate income and pensions.

#### 3.2.3. Controlled Variables

Drawing from existing literature, the study also includes several control variables: gender (Gender), age (Age), education level (Education), marital status (Married), household registration type (Household_t), number of children (Offspring), annual income (Income), pension status (Pension), and the impact of COVID-19 (COVID1, COVID2). As detailed in Table 1, the sample has an approximately equal distribution of men and women, with over 50% having a secondary school education or below. The rural population accounted for 73.53% of the sample, and more than 80% receive pension income. Under the influence of COVID-19, older people reported significantly reduced outdoor activity. The descriptive statistical results of the continuous variables in Table 2 show that the average age of the 9417 participants is 68, ranging from 60 to 108, indicating a relatively large proportion of younger seniors. On average, participants have 3 children, reflecting the higher birth rates in rural areas, unaffected by the family planning policy. COVID-19-related travel restrictions meant that older people, on average, did not leave home for 20 days, with the longest recorded period being 240 days, aligned with China’s prevention and control policy at that time. Prolonged inactivity and limited social engagement pose great challenges to their physical health.

Existing studies have pointed out that older people with stable economic resources are more willing to engage in social activities, which can improve their health through exercise and other means [20]. Therefore, this study took social participation (Social) and physical activity (Exercise) as mediating variables to explore how financial support influences the physical health of older people. In this study, social participation refers to whether the respondents often engaged in social activities in the past month, such as visiting, dancing, going to school, attending training, participating in club activities, etc., which was measured by binary variables. Physical activity refers to whether the respondent regularly exercises for more than 30 min per week, and if so, it indicates that the participant has a high frequency of exercise participation, which was also measured by the binary variables.

As can be seen from Table 1, the number of older people who often participate in physical exercise is basically the same as those who do not. In terms of social interaction, 45.73% of older people socialize frequently, which is slightly lower than those who do not.

### 3.3. Model Construction

To test the impact of financial support on the physical health of older people, ordered logit was used to construct the baseline regression model of this study according to the characteristics of the dependent variables:

(1) $$Y_{i}=\beta_{0}+\beta_{1}X_{1}+\sum \beta_{k}Z_{i}+\varepsilon_{i}$$

(2) $$Y_{i}=\beta_{0}+\beta_{1}X_{2}+\sum \beta_{k}Z_{i}+\varepsilon_{i}$$

where

Y is the explained variable, which represents the subjective physical health status of the i-th older person;

X includes explanatory variables X_1_ (property income of older people) and X_2_ (financial support of their children);

Z includes control variables, such as gender, age, marital status, education, etc;

ε is a random disturbance term following the logistic distribution;

β_0_ is the intercept term, while β_1_ and β_k_ are parameters to be estimated.

For the action path analysis, this study further explores both the direct impact of financial support on the physical health of older people and the indirect impact of social participation and physical activity with an intermediary effect model. The mediation test procedure mainly includes the following:

- Construct the regression equation of the influence of financial support on the physical health of older people (3);
- If the regression coefficient of financial support is significant, then the regression Equation (4) of the influence of financial support on the intermediary variables and the regression Equation (5) of the influence of financial support and intermediary variables on the physical health of older people are constructed to test whether the intermediary effect exists.

If the coefficient of financial support in Equations (4) and (5) is significant and the coefficient of the mediating variable in Equation (5) is also significant, then it is a partial mediating effect. If the coefficient of financial support in Equation (4) is significant, but only the coefficient of the intermediary variable in Equation (5) is significant while the coefficient of financial support is not, then it is a complete intermediary effect. Based on this, the specific model is as follows:

(3) $$Y_{i}=\alpha_{0}+\alpha_{1}X_{i}+\sum \alpha_{k}Z_{i}+\varepsilon_{i}$$

(4) $$M_{i}=\beta_{0}+\beta_{1}X_{i}+\sum \beta_{k}Z_{i}+\varepsilon_{i}$$

(5) $$Y_{i}=\gamma_{0}+\gamma_{1}X_{i}+\gamma_{2}M_{i}+\sum \gamma_{k}Z_{i}+\varepsilon_{i}$$

In the above equation, M is the mediating variable, including social participation and physical activity. While other variables in the formula are consistent with the previous definitions.

This study focuses on the estimate of coefficient α_1_/β_1_/γ_1_/γ_2_ in the model, where

α_1_ measures the net effect of financial support on the physical health of older people;

β_1_ measures the impact of financial support on the intermediary variables;

γ_1_ measures the direct impact of financial support on the physical health of older people;

γ_2_ measures the impact of the intermediary variables on the physical health of older people.

## 4. Results

### 4.1. Basic Regression

Table 3 shows the baseline regression results of the sequential regression test. Columns (1) and (2) are test results showing the impact of property ownership (Property) on the physical health of older people (Health), while Columns (3) and (4) display the impact of children’s financial support (CFS) on the physical health of older people (Health). Columns (1) and (3) report the absence of control variables. By contrast, after the addition of control variables, Pseudo R2 increased slightly, indicating an improved overall fit of the model.

The results in Column (2) reveal that property ownership is positively associated with the physical health of older people and is significant at the 1% level. In other words, the higher the property income, the better the physical health of older people, which is consistent with previous research findings. As can be seen from Table 1, there are relatively few older people with property income, and there are still great development prospects in promoting the health level of older people through improving property income. On the contrary, it can be seen from the results in Column (4) that the financial support of children is significantly negatively associated with the physical health of older people. That is, the more financial support provided by children, the worse the physical health of older people, which challenges the results of previous studies. First, older adults are more likely to have daily care of their children to meet their emotional needs rather than relying solely on the economy to meet them [21]. Second, older persons who have financial support from their children are either disabled and need financial support from their children to meet their medical needs [22], or they do not have independent earning ability and are very dependent on their children, which will have an impact on their view of money. They will only use the money for daily expenses rather than fitness and leisure.

In addition, the estimated results of relevant control variables are basically in line with expectations. Older men generally rated their physical health higher than their female counterparts. Age is negatively correlated with health, as physical health tends to decline with age. Education level showed a positive association with health, indicating that higher education enhances health awareness among older people. Urban residents had better health outcomes than rural residents, likely due to the relatively superior medical and material conditions in the city. Higher annual income also correlated with better health, supporting Maslow’s hierarchy of needs theory, where economic abundance allows focus on higher-level needs, such as health. During the prevalence of COVID-19, prolonged confinement led to reduced opportunities for social and physical activity, adversely affecting the physical health of older people.

### 4.2. Robustness Test

To ensure the accuracy of the research results, a robustness test was carried out by changing the explanatory variables, narrowing the sample scope, and modifying the control variables. In addition, in order to eliminate the impact of the 2020 epidemic, we also conducted a robustness test using the 2018 data.

Firstly, the independent variable was converted into a binary variable to directly reflect whether older people received family financial support, allowing a direct comparison of the physical health of those with and without such support. Secondly, given the central role of family financial support in this study—particularly the financial contribution of children—the analysis excluded childless older adults, as their inclusion could bias the measurement of family financial support. The results of this robustness test are shown in Columns (3) and (4) of Table 4. Thirdly, since household registration may not clearly distinguish older people from urban and rural areas, the variable “Household_t” was replaced by the variable “urban”, which categorizes residence types as a city center or town center/combination zone between urban and rural/village/special area. This adjustment accounts for shifts in living conditions and lifestyle choices, such as urban residents opting for rural retirement or rural residents moving to urban areas for better healthcare. By redefining the distinction between urban and rural areas, the analysis better reflects the relationship between financial support and physical health according to the actual residence of older people. Finally, we used 2018 data to eliminate the impact of epidemic factors on older adults’ health, financial support, and related control variables. Since property income and children’s economic support were not clearly divided when the data of 2018 was obtained, financial support was uniformly used as an independent variable in this part.

To sum up, the robustness test results are shown in Table 4, which confirms that the model estimation structure in this study is robust and reliable.

### 4.3. Heterogeneity Analysis

Previous studies have shown that the effects of financial support on the physical health of older people are heterogeneous by age and place of residence. Therefore, this study carried out a heterogeneity analysis based on these factors. As shown in Table 5, property ownership has a more pronounced impact on the physical health of older people aged 70 and above and those living in rural areas. In addition, children’s financial support is significant only for those aged 70 and over. Based on this, H3a and H3b are verified.

### 4.4. The Mechanism of Financial Support on the Physical Health of Older People

The mechanism linking financial support and the physical health of older people is not yet clear, but relevant studies have suggested a strong correlation between social participation (Social), physical participation (Exercise), and the physical health of older people. To further investigate, this study selected social participation and physical activity as mediators to verify the mechanism of action. Using the stepwise regression method, this study explored the intermediary path of property ownership and children’s financial support, respectively.

**Figure 1:**
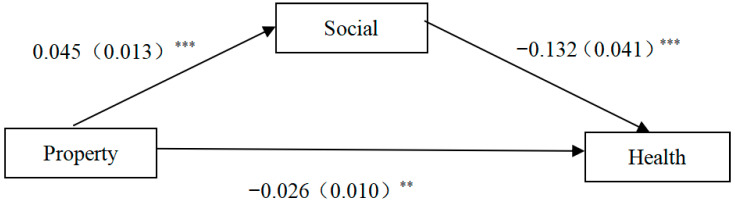
Pathways of the impact of property on the physical health of older adults. Note: (1) ** *p* < 0.05; *** *p* < 0.01. (2) Robust standard error for t-statistics in parentheses.

Figure 1 clearly shows the effect of property ownership on the physical health of older people. The results reveal a significant positive correlation between property ownership and social participation, while both property ownership and social participation are positively correlated with the physical health of older people. The indirect effect of social participation on the physical health of older people is significant at the 1% level. In other words, social participation has a significant mediating effect between property ownership and the physical health of older people, and the mediating effect is 0.006, which is significant at the 1% level.

**Figure 2:**
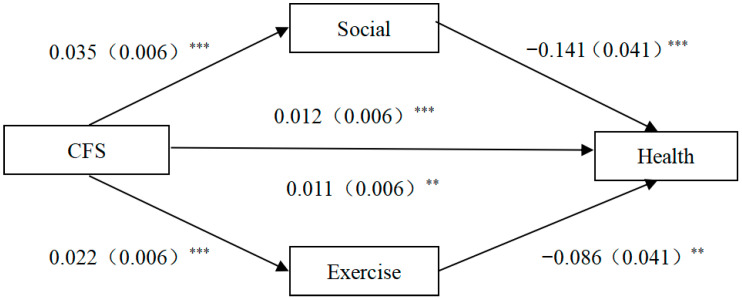
Pathways of the impact of CFS on the physical health of older adults. Note: (1) ** *p* < 0.05; *** *p* < 0.01. (2) Robust standard error for t-statistics in parentheses.

Figure 2 depicts the impact of children’s financial support on the physical health of older people. The analysis shows a significant positive correlation between children’s financial support and both social and sports participation. Children’s financial support, social participation, and physical activity are all significantly related to the physical health of older people. Notably, social participation has a significant mediating effect between children’s financial support and the physical health of older people, with a mediating effect of 0.005, which is significant at the 1% level, whereas the indirect impact of physical activity on the physical health of older people is significant at the 5% level, that is, the mediating effect of physical activity is significant, and the mediating effect is 0.002, which is smaller than that of social participation.

## 5. Discussion

### 5.1. Summary

This study used data from the 2020 CHARLS to analyze the impact of financial support on the physical health of older people through heterogeneity analysis and mediation tests, aiming to identify factors affecting their health. The results show that when controlling variables and urban fixed effects are included, both property ownership and children’s financial support have significant effects on the physical health of older people, which is consistent with some previous research results [6,23].

In terms of control variables, the self-rated physical health status of aging females was significantly worse than that of aging males. This disparity aligns with other research, partly because women are generally more emotional and more concerned about their own health, potentially amplifying their pain perception [24]. Meanwhile, relevant studies have also pointed to higher social happiness among aging males, which also has a certain positive impact on their physical health [25]. Regarding household registration categories, older people with urban household registration report significantly better health than their rural counterparts. This reflects the substantial disparity between urban and rural areas in China, where urban residents benefit from superior medical facilities and higher living standards. What is more, rural older people often lack nearby family support due to their children working as migrant laborers, while the higher socioeconomic status of their urban counterparts contributes to better physical health [26]. Additionally, the effect of annual personal income was also consistent with expectations: The higher the annual personal income, the better the physical health of older people. The annual income level is usually closely related to education and social status. Older people with higher education and higher income have better healthcare awareness, which leads to more investment in healthcare, physical exercise, and expensive wellness products [27]. The COVID-19 pandemic has further influenced older people’s health outcomes, as they were advised to minimize outings to comply with relevant prevention and control regulations [28]. The results show that the more time spent at home, the worse the physical health of older people. Primarily, reduced outings may diminish their normal social interactions. Secondarily, it may also lead to fewer opportunities for outdoor activities, such as chess, walking, tai chi, and other low-intensity exercises. All this hampers their physical and mental health [29].

The heterogeneity analysis shows that the impact of financial support on the health of older people was significantly different with respect to age and urban–rural dwellings. Notably, the impact of property ownership on physical health is positive for both age groups, yet it is more pronounced among those aged 70 and above. Conversely, children’s financial support is only significant for those over 70, exhibiting a significantly negative correlation. This may stem from the increasing dependence on property-based financial income as individuals age. As people reach 70, their physical capabilities decline, the prevalence of various chronic diseases escalates [30], and the ability to earn a living diminishes [31]. With the rising number of older people over 70, the corresponding medical expenditure also surges. In the context of Chinese traditional culture, children are expected to raise the alimony of their parents to ensure superior healthcare, thereby fulfilling their filial duties [32]. Consequently, an increase in children’s financial support may indicate a higher likelihood of chronic or severe diseases among older adults, suggesting an inverse relationship between children’s financial support and the health of older people. On the contrary, a predominance of property income indicates relative health and reduced reliance on children’s financial and material support from their children. In terms of urban and rural heterogeneity, only property ownership has a significant impact on the health of older people, with rural seniors experiencing a more substantial effect. This could be attributed to the deepening of China’s rural revitalization strategy, which galvanized the rural economy and rural industries. Many rural residents have used their own fixed assets, such as real estate and fields, to build homesteads, tourist resorts, and farm stays. Meanwhile, agricultural mechanization and other measures have also effectively guaranteed the economic benefits of rural residents [33]. Therefore, rural seniors are more dependent on fixed assets, which can affect their living standards for a long period of time. On the contrary, urban seniors, aside from fixed asset income, often meet their living needs through personal skills or pensions, demonstrating less dependence on fixed assets.

The results of the mediation analysis indicate a positive correlation between both property ownership and children’s financial support with the social and sports participation of the old, which is consistent with previous research findings [6]. Family financial assistance can enhance older people’s sense of pride, self-confidence, and overall happiness [34]. When their basic needs are satisfied and they have a sufficient economic surplus, they tend to engage in necessary social interactions and exercise in outdoor settings such as communities, squares, and river walks [35]. These activities not only fulfill their need for peer communication but also serve as a means to convey their social and economic status, signaling a satisfactory standard of living that may even be superior in certain aspects. In addition, the results also show that both social and sports participation significantly improved the physical health of older people. While physical activity directly improves health, social communication indirectly contributes by fostering self-esteem and other psychological emotions, which encourage older people to maintain exercise routines, work and rest patterns, and a balanced diet, thereby strengthening their immunity and health and making them appear superior to others [36]. In contrast, the effect path of property ownership on the physical health of older people operates mainly through social participation, whereas children’s financial support impacts physical health through both social and sports participation. This is attributed to the fact that discussions among Chinese older adults often revolve around their children’s development, such as their careers, income, assets, families, and social status [37]. At the same time, exercise is an important medium of social communication for older people, giving them excellent opportunities to create a positive sports environment and disseminate information on their children’s achievements. Therefore, children’s financial support influences the physical health of older people through both social and sports participation pathways.

### 5.2. Policy Implications

From the conclusions of this study, the following policy implications can be summarized: First, older people are encouraged to re-enter the labor market and engage in more meaningful occupational activities. The results show that the wealth and income that older people own can have a positive impact on their health. In this context, encouraging capable older people to remain engaged in the workforce would bring dual benefits to individuals and society. It not only increases their personal income, helping them maintain a higher standard of life and better manage living and healthcare expenses [38] but also contributes to their physical and mental well-being through social interaction and daily errands. The same applies to older people with no income. If older people without income, who maintain their living through social security, can realize their personal value through re-employment, they can not only relieve the pressure of life but also have a positive impact on their health from a physical and psychological perspective. Concurrently, the re-employment of older people can alleviate the financial burden of the government and reduce the strain on pension and social security systems.

Second, older people should be encouraged to actively engage in social and sports activities. According to the findings, such engagement is instrumental in maintaining good health. After retirement, participating in social activities can mitigate the risks of loneliness and social isolation by establishing new social connections with peers and those sharing similar interests, reducing loneliness and depression. Active participation in physical exercise, such as tai chi, square dance, martial arts, walking, etc., can help older people maintain good physical fitness, improve cardiopulmonary function, strengthen muscles, and enhance balance, preventing common age-related health problems, such as fractures, arthritis, cardiovascular, and cerebrovascular diseases. For the older adult group without income, it is still difficult to improve their health through sports and social participation. They still need to meet their basic needs of life and improve their health by referring to previous ways of re-employment. At the same time, the process of older adults participating in work is also a process of participating in social interaction and sports. Firstly, this is because work requires dealing with colleagues, which can promote the social integration of older adults. Secondly, compared with the sedentary and less active older people at home, participating in work can effectively improve their daily exercise, which is of great benefit to their health levels [39].

Third, a community-based home care model is advocated. The results show that financial support is closely related to the physical health of older people, while children’s financial support can potentially diminish the health of older people, mainly due to the conflict between career demands and filial duties. When children provide substantial financial support, they may have less time to take care of their parents, which has an adverse impact on the physical and mental health of older people. Therefore, integrating the functions of traditional family care with the advantages of home care, the new model of community-based home care presents a significant approach to balancing the relationship between children and older people. Establishing day care centers within communities, regularly sending professionals to provide physical examination services, and educating family members in basic nursing skills can offer older people family-like care without the need to leave their residences. This model allows their children to work with peace of mind, sustain the family’s source of income, and provide the necessary material living conditions for older people.

### 5.3. Strengths

This study offers several methodological and analytical strengths. First, it examined the mediating role of social and sports participation in the relationship between financial support and the physical health of older people. This not only complements previous research but also provides new evidence to understand the interplay between physical health, social engagement, physical activity, and financial support among older people. Second, the study used the most recent data from CHARLS to estimate the impact of financial support on the physical health of older people. This helps to directly reflect the factors that affect the physical health of older people in the wake of COVID-19. Third, this study’s analysis of heterogeneity across age groups and urban–rural divides provides a nuanced understanding of the relationship between financial support and the physical health of older people.

### 5.4. Limitations

The shortcomings of this study mainly include the following aspects: First, this study did not accurately measure the physical health status of older adults according to objective indicators. Objective and subjective health indicators complement each other and can be used as a robustness test to support the research results. In the future, objective indicators, such as the BMI index and the ADL index, can be used to measure the physical health level of older people. Second, the financial support in this study only includes property ownership and children’s financial support. This will be further subdivided in the future to dissect the specific contributions of various forms of financial support to the health outcomes of older people. Third, this study focused solely on the physical health of older people, neglecting to measure the mental and social health of overall health quality. However, because the key of this study is to study the physical health of older adults rather than the overall health status, such as the quality of life, it is not necessary to include the dimensions of mental health and social health in this study. Based on this, in order to further explore the relationship between economic support and quality of life for older adults in the future, the dimensions of mental health and social health will be introduced.

## 6. Conclusions

Using the micro survey data of CHARLS2020, this study empirically analyzed the influence of financial support on the physical health of older people and its mediating mechanism through the ordered logistic regression model. The results show that property ownership significantly enhances the physical health of older people, whereas children’s financial support exerts a notably negative influence. These outcomes were substantiated through robustness testing. Heterogeneity analysis reveals that property ownership has a more pronounced positive effect on the physical health of older people aged 70 and above and those residing in rural areas. Similarly, the negative impact of children’s financial support on the physical health of older people is particularly evident in those aged 70 and above. The intermediary analysis suggests that the promotion effect of property ownership on the physical health of older people is mainly achieved through increased social participation. In contrast, the influence of children’s financial support on the health of older people operates through two primary pathways: social participation and physical activity. The main contributions of this study are three-fold: first, we distinguished the effects of different types of financial support on the health of the older people; second, we explored the mediating role of social participation and physical activity; and third, we further highlighted the differences between different ages and urban and rural older people.

## Figures and Tables

**Table 1:** Descriptive statistics of categorical variables.

Variable	Description	Frequency	Percent
Health	Very good	940	9.98%
	Good	1075	11.42%
	Fair	4699	49.90%
	Poor	2004	21.28%
	Very poor	699	7.42%
Property_s	No	9030	95.89%
	Yes	387	4.11%
CFS_s	No	3300	35.04%
	Yes	6117	64.96%
Exercise	No	4473	47.50%
	Yes	4944	52.50%
Social	No	5111	54.27%
	Yes	4306	45.73%
Gender	Male	4575	48.58%
	Female	4842	51.42%
Education	Uneducated (illiterate)	2749	29.19%
	Did not finish primary school	2196	23.32%
	Graduated from private school	17	0.18%
	Graduated from primary school	1998	21.22%
	Graduated from junior high school	1513	16.07%
	Graduated from high school	575	6.11%
	Graduate from technical secondary school	223	2.37%
	College graduate	88	0.93%
	Bachelor’s degree	53	0.56%
	Master’s degree	3	0.03%
	Graduate with a PhD	2	0.02%
Married	Unmarried	57	0.61%
	Married	9360	99.39%
Household_t	Rural	6924	73.53%
	Urban	2493	26.47%
Pension	No	1821	19.34%
	Yes	7596	80.66%
COVID2	Increased greatly	41	0.44%
	Increased slightly	37	0.39%
	No changed	3930	41.73%
	Decreased slightly	825	8.76%
	Decreased greatly	4584	48.68%
Urban	City center or town center	2160	22.94%
	Combination zone between urban and rural	998	10.60%
	Village	6252	66.39%
	Special area	7	0.07%

**Table 2:** Descriptive statistics of continuous variables.

Variable	Observation	M	SD	Min	Max
Property	9417	0.368	1.806	0	12.206
CFS	9417	5.186	3.945	0	12.255
Age	9417	68.346	6.250	60	108
Offspring	9417	2.841	1.373	0	10
Income	9417	1.153	3.041	0	11.695
COVID1	9231	20.233	30.333	0	240

**Table 3:** Baseline regression results.

Variables	Health			
	(1)	(2)	(3)	(4)
Property	−0.034 *** (0.010)	−0.027 *** (0.010)		
CFS			0.016 *** (0.005)	0.010 ** (0.006)
Gender		0.166 *** (0.044)		0.162 *** (0.044)
Age		0.005 (0.004)		0.005 (0.004)
Education		−0.017 (0.013)		−0.020 (0.013)
Married		−0.037 (0.302)		−0.067 (0.302)
Household_t		−0.182 *** (0.055)		−0.181 *** (0.055)
Offspring		0.014 (0.019)		0.006 (0.019)
Income		−0.070 *** (0.007)		−0.070 *** (0.007)
Pension		−0.066 (0.056)		−0.071 (0.056)
COVID1		0.002 *** (0.001)		0.003 *** (0.001)
COVID2		0.030 (0.022)		0.027 (0.022)
City	Controlled	Controlled	Controlled	Controlled
Pseudo R^2^	0.017	0.025	0.017	0.025
N	9417	9231	9417	9231

Note: (1) ** *p* < 0.05; *** *p* < 0.01. (2) Robust standard error for t-statistics in parentheses.

**Table 4:** Robustness test results.

Variables	Alternate Explanatory Variable		Narrow Down the Sample		Change Control Variable		Using 2018 Data
	(1)	(2)	(3)	(4)	(5)	(6)	(7)
Property			−0.027 ** (0.011)		−0.025 ** (0.011)		
CFS				0.010 * (0.006)		0.012 ** (0.006)	
Property_s	−0.248 *** (0.095)						
CFS_s		0.099 ** (0.046)					
Financial support							−0.438 *** (0.166)
Urban					0.118 *** (0.029)	0.123 *** (0.029)	
Controlled variables	Controlled	Controlled	Controlled	Controlled	Controlled	Controlled	Controlled
City	Controlled	Controlled	Controlled	Controlled	Controlled	Controlled	Controlled
Pseudo R^2^	0.025	0.025	0.025	0.025	0.025	0.025	0.032
N	9231	9231	9117	9117	9231	9231	5887

Note: (1) * *p* < 0.1; ** *p* < 0.05; *** *p* < 0.01. (2) Robust standard error for t-statistics in parentheses.

**Table 5:** Heterogeneity test results.

Variables	Age		Household_t	
	(1) 60–69 Years Old	(2) Age 70 and Older	(3) Urban	(4) Rural
Property	−0.021 * (0.012)	−0.053 ** (0.024)	−0.007 (0.018)	−0.053 *** (0.014)
CFS	0.008 (0.007)	0.018 * (0.010)	0.013 (0.011)	0.010 (0.007)
Controlled variables	Controlled	Controlled	Controlled	Controlled
City	Controlled	Controlled	Controlled	Controlled
Pseudo R^2^	0.028	0.034	0.040	0.028
N	5940	3291	2466	6765

Note: (1) * *p* < 0.1; ** *p* < 0.05; *** *p* < 0.01. (2) Robust standard error for t-statistics in parentheses.

## Data Availability

This study utilizes data from the China Health and Retirement Longitudinal Study (CHARLS) database (available at https://charls.pku.edu.cn/, accessed on 19 June 2023).

## Supplementary Materials

The following supporting information can be downloaded at: https://www.mdpi.com/article/10.3390/healthcare13101163/s1, Table S1: Descriptions of variables.
