# Supplementary material for: Association Between Financial Support and Physical Health in Older People: Evidence from CHARLS Data
Source: Healthcare (Basel). 2025 May 16;13(10):1163. doi: 10.3390/healthcare13101163 (PMC12111041; doi:10.3390/healthcare13101163)
Supplement: Supplementary file 1 [file healthcare-13-01163-s001.zip › healthcare-3563609-supplementary.pdf]

Table S1. Descriptions of variables.

Variable	Description	Coding values
Dependent variable		
Health	Elderly people's evaluation of their own physical health	Measured on a scale of 1 (very good) to 5 (very poor)
Independent variable		
Property	The logarithm of the property rental amount of the respondent	Measured in Chinese yuan
Property_s	Whether the interviewee has any property for rent	Yes=1, no=0(Calculated by Property)
CFS	The logarithm of the financial support of the respondent's children and relatives	Measured in Chinese yuan
CFS_s	Whether the respondent has obtained financial support from children and relatives	Yes=1, no=0(Calculated by CFS)
Intermediate variable		
Exercise	Whether the respondent regularly exercised for more than 30 minutes a week	Yes=1, no=0
Social	In the past month, whether the respondent often socialized, such as visiting, dancing, going to school, attending training, participating in club activities, etc	Yes=1, no=0
Controlled variable		
Gender	Gender	Male=1, female=2
Age	Age	Measured in years
Education	Educational level	1. Uneducated (illiterate); 2. Did not finish primary school; 3. Graduated from private school; 4. Graduated from primary school; 5. Graduated from junior high school; 6. Graduated from high school; 7. Graduate from technical secondary school (including secondary normal school and vocational high school); 8. College graduate; 9.

		Bachelor degree; 10. Master's degree; 11. Graduate with a PhD
Married	Martial status	Married=1, unmarried=0
Household_t	Household registration type	Urban=1, rural=0
Offspring	Number of surviving children	
Income	The logarithm of an individual's annual income	Measured in Chinese yuan
Pension	Whether the respondent has a pension or pension	Yes=1, no=0
COVID1	What is the longest time the respondent has not been outside because of COVID-19	
COVID2	How has the time spent away from home for COVID-19 changed	1. Increased greatly; 2. Increased slightly; 3. No changed; 4. Decreased slightly; 5. Decreased greatly
Urban	Type of residence of the respondent	1. City Center or Town Center; 2. Combination Zone Between Urban and Rural; 3. Village; 4. Special area
